# Molecular mechanisms of cisplatin cytotoxicity in acute promyelocytic leukemia cells

**DOI:** 10.18632/oncotarget.5754

**Published:** 2015-10-15

**Authors:** Sanjay Kumar, Paul B. Tchounwou

**Affiliations:** ^1^ Cellomics and Toxicogenomics Research Laboratory, NIH/NIMHD-RCMI Center for Environmental Health, College of Science, Engineering and Technology, Jackson State University, Jackson, Mississippi 39217, USA

**Keywords:** cisplatin, APL cell line, cell cycle modulation, AP-1 and p53

## Abstract

Cis-diamminedichloroplatinum (II) (cisplatin) is a widely used anti-tumor drug for the treatment of a broad range of human malignancies with successful therapeutic outcomes for head and neck, ovarian, and testicular cancers. It has been found to inhibit cell cycle progression and to induce oxidative stress and apoptosis in acute promyelocytic leukemia (APL) cells. However, its molecular mechanisms of cytotoxic action are poorly understood. We hypothesized that cisplatin induces cytotoxicity through DNA adduct formation, oxidative stress, transcriptional factors (p53 and AP-1), cell cycle regulation, stress signaling and apoptosis in APL cells. We used the APL cell line as a model, and applied a variety of molecular tools to elucidate the cytototoxic mode of action of cisplatin. We found that cisplatin inhibited cell proliferation by a cytotoxicity, characterized by DNA damage and modulation of oxidative stress. Cisplatin also activated p53 and phosphorylated activator protein (AP-1) component, c-Jun at serine (63, 73) residue simultaneously leading to cell cycle arrest through stimulation of p21 and down regulation of cyclins and cyclin dependent kinases in APL cell lines. It strongly activated the intrinsic pathway of apoptosis through alteration of the mitochondrial membrane potential, release of cytochrome C, and up-regulation of caspase 3 activity. It also down regulated the p38MAPK pathway. Overall, this study highlights the molecular mechanisms that underline cisplatin toxicity to APL cells, and provides insights into selection of novel targets and/or design of therapeutic agents to treat APL.

## INTRODUCTION

Acute promyelocytic leukemia (APL) is a blood cancer usually formed through chromosomal translocation between chromosomes 15 and 17 inside the bone marrow during blood formation. It accounts for approximately 10% of all acute myeloid leukemia (AML) [1]. About 1500 APL affects people in USA every year, with case fatality rate of about 1.2%. Arsenic trioxide (ATO) and all trans retinoic acid (ATRA) combination is the most successful treatment of all age group of APL patients with maximum survival rate [2]. Previous study has shown that ATO performed complete remission with high survival rate without ATRA combination in APL patients [3]. It was also reported that ATO alone treated to APL patients achieved high rate of 5-years disease free survival (DFS) and an overall survival (OS) in Phase II clinical trial study [4]. Resistance to this conventional chemotherapy has been reported in APL patients [5]. Hence, there is a need for new therapeutic strategies to enhance the clinical outcomes of APL patients. Cisplatin is an anti-tumor drug [6], highly used in treatment of different kinds of human malignancies including bladder, head and neck, lung, ovarian, and testicular cancers [7]. It has been reported that cisplatin combination with ATO enhances cytotoxicity in head and neck squamous cell carcinoma cell [8] and small cell lung cancer [9]. It has also been reported that cisplatin may be a good combination for ATO to overcome drug resistance and enhance cytotoxicity in APL cells. Some studies have shown that cisplatin causes DNA cleavage and cell death in APL cells by modulating c-jun expression and PKC signaling [10]. It also induces apoptosis in HL-60 cells through BCL_2_ down regulation and activation of BCL_2_L12 expression [11], oxidative stress and inhibition of cell cycle progression [12]. Other studies have reported that cisplatin interacts with DNA, RNA and proteins, and causes cytotoxicity. It produces cytotoxicity through formation of DNA-adduct, inducing oxidative stress, inhibition of DNA synthesis, modulation of cell cycle and induction of apoptosis [6, 10, 12, and 13]. Cisplatin has also been found to induce cytotoxicity through activation of caspase 3 in CEM cells, a human T leukemia [14] and formation of DNA adduct in mouse leukemia L1210 cells [15].

Only few studies have focused on cisplatin - induced cytotoxicity, oxidative stress and apoptosis in APL cells. For the first time, we propose a detailed molecular mechanism of the cytotoxic action in APL cells, based on the novel findings that cisplatin induces cell proliferation inhibition, DNA- adduct formation, activation of transcriptional factors, oxidative stress, cell cycle arrest and intrinsic pathway of apoptosis in APL cells.

## RESULTS

### Cisplatin inhibits APL cells proliferation and promotes DNA adduct formation

**Figure 1:**
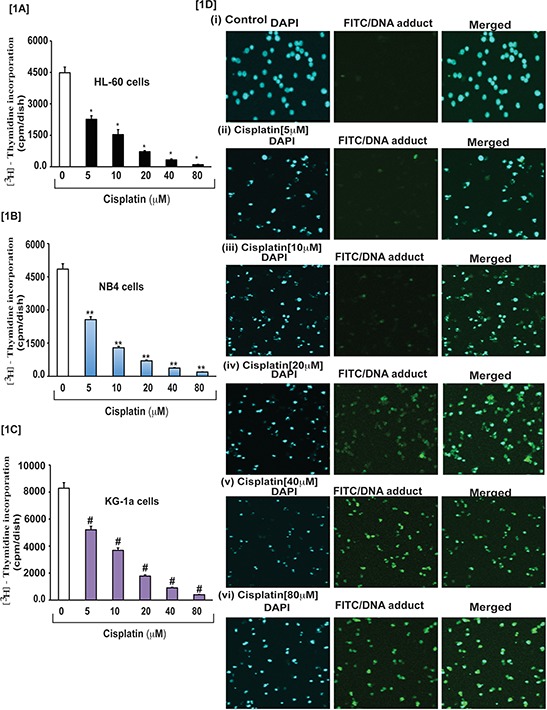
Cisplatin inhibits growth and induced formation of DNA-adduct in APL cells APL cells (HL-60, NB4 and KG-1a) were exposed to various concentrations (0, 5, 10, 20, 40, and 80 μM) of cisplatin for 24 hours and further incubated for 24 hours with tritium labeled thymidine. After incubation, cells were harvested by centrifugation and counted using liquid scintillation analyzer as described in the material and method section. ^3^H-methyl thymidine incorporation was expressed as cpm/dish. Data represent the means of three independent experiments ± SDs. Highly statistically significant decreases (*p* < 0.01) in cell proliferation were observed in all cisplatin treated APL cells including HL-60 [1A], NB4 [1B] and Kg-1a [1C] cells. This reduction in cell growth was concentration-dependent. Cisplatin –induced formation of DNA adduct was assessed by immunocytochemistry and confocal microscopy analysis as described in the material and methods section. APL cells were exposed to various concentrations of cisplatin for 48 hours and performed immunocytochemistry as well as confocal microscopy using FITC filter to confirm DNA adduct formation. The results showed that cisplatin caused a significant concentration -dependent increase in DNA-adduct formation in APL cells [1D (i-vi)].

To investigate the effect of cisplatin on APL cells growth, we conducted cell proliferation assay using methyl thymidine incorporation protocol. Hence, cells were treated with various concentrations (0, 5, 10, 20, 40, and 80 μM) of cisplatin and incubated with tritium labeled methyl thymidine for 21 hours. After incubation, the cells were harvested and radioactivity was measured using a liquid scintillation analyzer (Tri-carb 2700TR, Perkin Elmer). Radio activities were expressed as counts per minute (CPM) for each sample. Study results indicated that cisplatin inhibited significantly cells growth in a concentration - dependent manner in all three APL cell line (Fig. 1A–1C). We have also analyzed cisplatin-induced DNA adduct formation through immunocytochemistry and confocal imaging of control and cisplatin-treated APL cells. We found that cisplatin significantly (*p* < 0.01) induced DNA adduct formation in a concentration- dependent manner in APL cells [Fig. 1D (i-vi)].

### Cisplatin causes cytotoxicity in APL cells

**Figure 2:**
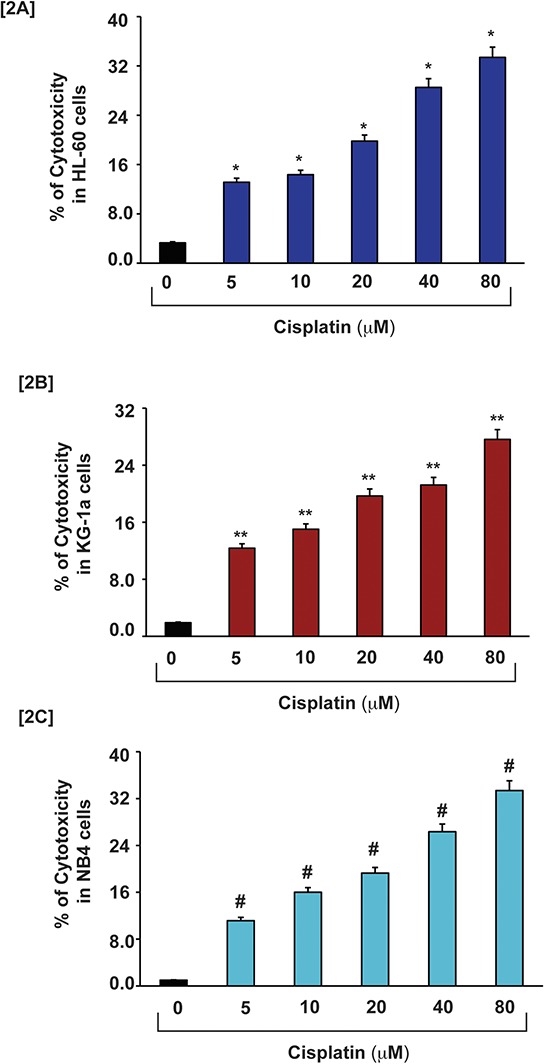
Cisplatin induces cytotoxicity in APL cells APL cells were exposed to various concentrations (0, 5, 10, 20, 40 & 80 μM) of cisplatin for 48 hours and LDH released in medium was measured using Promega non-radioactive cytotoxicity assay technical bulletin protocol. Then % cytotoxicity was calculated by dividing the levels of released LDH in treated cells over the total LDH released from control cells. Highly statistically significant increases (*p* < 0.01) in cytotoxicity were observed in all cisplatin treated APL cells including HL-60 [2A], KG-1a [2B] and NB4 [2C] cells in a concentration - dependent fashion.

To investigate the cytotoxic effect of cisplatin with APL cells, we exposed three APL cell lines (HL-60, KG-1a and NB4 cells) for 48 hours to various concentrations of cisplatin in triplicate, and assayed LDH released in the medium by measuring absorbance at 490 nm. Our results show that cisplatin induces cytotoxicity in a concentration - dependent manner. Significant differences (*p* < 0.01) were observed in all three APL cell lines between cisplatin - treated cells and untreated cells (control). Significant increases in % of cytotoxicity were observed in all three cell lines including HL-60, KG-1a and NB4 cells as shown in [Fig. 2A–2C].

### Cisplatin induces oxidative stress and clastogenic effect

**Figure 3:**
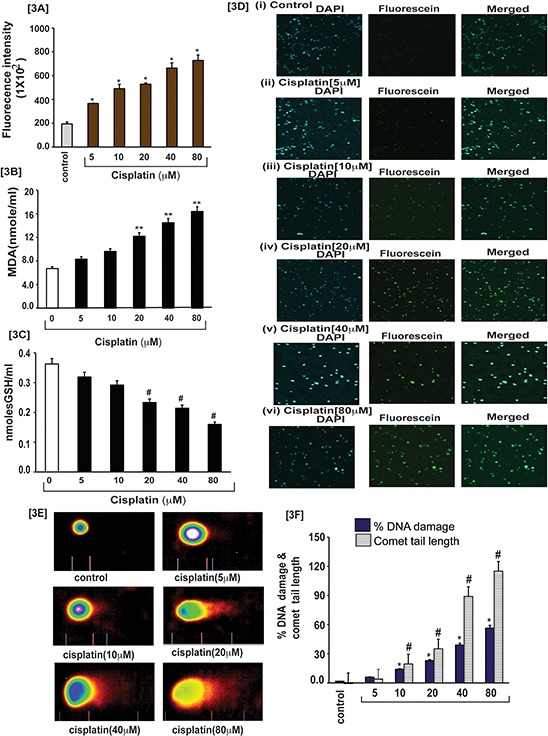
Cisplatin induces oxidative stress and clastogenic effect in APL cells Cisplatin induced oxidative stress and clastogenic effect in APL cells through generation of reactive oxygen species (ROS) and formation of DNA adduct. APL cells were exposed to various concentrations (0, 5, 10, 20, 40, and 80 μM) of cisplatin for 48 hours and further treated 30 min with dichlorofluorescein diacetate (DCFDA). After incubation, ROS released was measured through measuring DCF fluorescence intensity by spectrofluorementry. Data represent the means of three independent experiments ± SDs (*P < 0.01) [3A]. APL cells were exposed to various concentrations of cisplatin and lipid peroxidation one product, malondialdehyde (MDA) formation measured by spectrophotometry. Data represent the means of three independent experiments ± SDs (**P < 0.01) [3B]. APL cells were exposed to various concentrations of cisplatin and GSH level was measured by spectrophotometry. Data represent the means of three independent experiments ± SDs (#*P* < 0.05) [3C]. APL cells were exposed to various concentrations of cisplatin and DNA damage analyzed by TUNEL assay [3D (i-vi)]. APL cells were exposed to various concentrations of cisplatin and DNA damage was analyzed by alkaline gel electrophoresis (Comet) assay and characterized by comet tail length and % DNA damage. Data represent the means of three independent experiments ± SDs % DNA damage (*P < 0.01) and comet tail length ($, *P* < 0.01) [3E-3F].

For searching the causative factor of cisplatin cytotoxicity in APL cells, we targeted cisplatin–induced reactive oxygen species (ROS) production and three biomarkers of oxidative stress. After exposure of APL cells to various concentrations of cisplatin for 48 hours, the cells were further incubated with dichlofluroscein diacetate (DCFDA) for 30 min and ROS production was measured by fluorescence (DCF) analysis at excitation (485 nm) and emission (520) using POLARstar Omega (Ortenberg, Germany). Three biomarkers of oxidative stress including lipid peroxidation, GSH level and DNA damage were also measured in both control and cisplatin treated APL cells. Our results indicated that cisplatin increased ROS production in a concentration - dependent manner [3A] and also stimulated lipid peroxidation as characterized by an increase in MDA formation and DNA damage and by reducing GSH content in APL cells significantly [Fig. 3B–3F]. Cisplatin exposure also produced a significant clastogenic effect through DNA damaging in APL cells, as shown in both in TUNEL assay [Fig. 3D] and comet assay [Fig. 3E–3F].

### Cisplatin modulates cell cycle regulation

**Figure 4:**
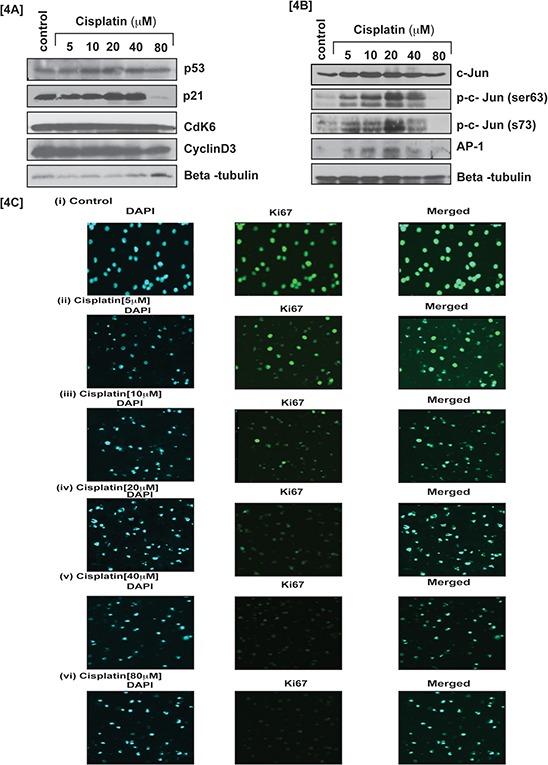
Cisplatin modulates cell cycle regulation by transcriptional factors Cisplatin induced cell cycle arrest at G1 checkpoint by stimulating transcription factors, p53 and phosphorylation of activator protein -1(AP-1) components in APL cells. APL cells were exposed to various concentrations (0, 5, 10, 20, 40 and 80 μM) of cisplatin for 48 hours and western blotting was performed for checking the expression level of cell cycle regulatory proteins, p53, p21, cdk6 & cyclin D3 [4A]. Transcriptional factor, AP-1 expression and their components, c-Jun phosphorylatin at ser 63 & Ser73 residue were checked by western blotting in both control and cisplatin treated APL cells [4B]. APL cells were exposed to various concentrations of cisplatin and the expression level of cell proliferation marker, Ki67 was assessed by immunocytochemistry and confocal imaging method. The results show a significant reduction in the expression level of Ki67 in cisplatin treated cells compared to control cells [4C (i-vi)].

To study cisplatin –induced oxidative stress and clastogenic (DNA damage) effect on APL cell cycle modulation, we exposed APL cells for 48 hrs to various concentrations of cisplatin and analyzed potent cell cycle regulatory transcriptional factor phosphorylation and expression by western blotting. Interestingly, stress transcriptional factors, activator protein-1 (AP-1) and p53 expression were stimulated in a concentration - dependent manner in cisplatin treated cells compared to untreated (control) cells. Significant up regulation was obtained for phosphorylated AP-1 component, c-Jun at serine (63, 73) residue [Fig. 4B], whereas cyclins and cyclin dependent kinases (cdks) expression was down regulated concentration - dependently in APL cells treated with cisplatin [Fig. 4A]. Cisplatin cytotoxicity also stimulated cell cycle proliferation inhibitor protein [inhibitor of cyclin dependent kinases (cdks)], p21 expression significantly in APL cells [Fig. 4A]. Our immunocytochemistry experiment also showed a significant reduction in ki67 (a biomarker of cell proliferation) expression cisplatin treated APL cells compared to control cells. Overall, Cisplatin exposure increased expression of p53, p21 and AP-1 (as well as their component, c-Jun residue phosphorylation) leading to cell cycle arrest, which was also confirmed by a significant concentration – dependent decrease in the expression of ki67 [Fig. 4A–4C (i-vi)].

### Cisplatin induces intrinsic pathway of apoptosis

**Figure 5:**
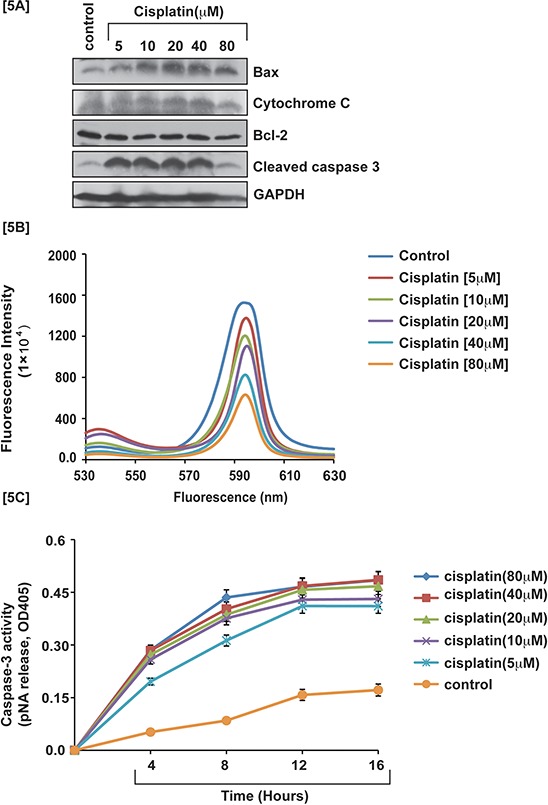
Cisplatin induces intrinsic pathway of apoptosis Cisplatin-induced cytotoxicity activated the intrinsic pathway of apoptosis in APL cells. APL cells were exposed to various concentrations (0, 5, 10, 20, 40 and 80 μM) of cisplatin for 48 hours and western blotting was performed to check the expression level of pro-apoptotic proteins (cytochrome C & Bax), caspase 3, and anti-apoptotic protein, Bcl-2 [5A]. APL cells were exposed to various concentrations of cisplatin and the mitochondria of all treated and control cells were isolated. Isolated mitochondria were treated with JC-1 dye for 10 min and mitochondrial membrane potential was assessed by spectrofluorometry [5B]. APL cells were exposed to various concentrations of cisplatin and caspase 3 activity was measured at different time points [5C].

**Figure 6:**
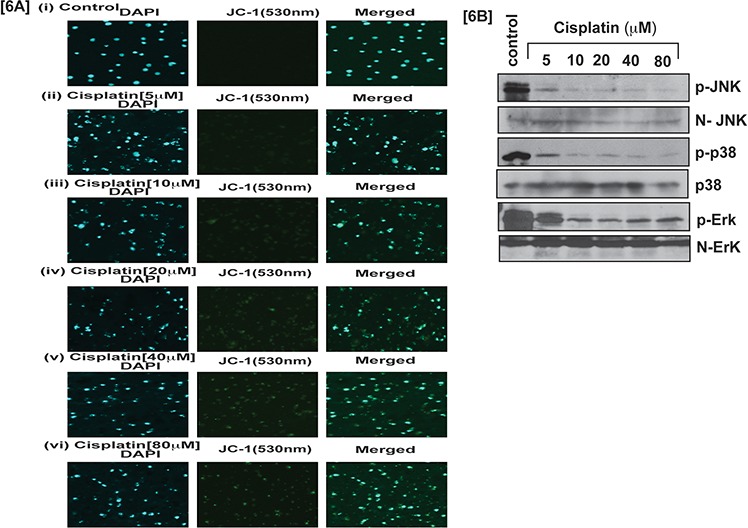
Cisplatin induces apoptosis and modulates stress signaling Cisplatin effect on JC-1 monomer expression and MAPK signaling pathway in APL cells was assessed. APL cells were incubated to various concentrations (0, 5, 10, 20, 40, and 80 μM) of cisplatin for 48 hours and mitochondria were isolated. Isolated mitochondria were treated with JC-1 dye 10 min, and immunocytochemistry and confocal imaging were performed to check the expression level of JC-1 monomer [6A (i-vi)]. APL cells were incubated to various concentrations of cisplatin and western blotting was performed to check the phosphorylation level of MAPK signaling molecules like JNK, p38 & Erk [6B].

Cisplatin induced p53 activation in APL cells caused an arrest in cell cycle progression mostly at the G1 checkpoint and forced the cells to undergo intrinsic pathway of apoptosis. We investigated, cisplatin - induced intrinsic pathway of apoptosis in APL cells by western blot analysis of the expression levels of proapoptotic proteins (e.g. Bax, cytochrome, and caspase 3) and anti-apoptotic protein (Bcl-2) by western blotting both in control and treated cells. Our results provided evidence that cisplatin stimulated the expression levels of pro-apoptotic proteins and also down regulated the expression levels of anti-apoptotic proteins in a concentration - dependent manner [Fig. 5A]. Cisplatin also induced oxidative stress and clastogenic effect leading to a change in mitochondrial membrane potential, and subsequent release of cytochrome C into the cytosol, which then activated caspase 3 in APL cells in a concentration - dependent manner. Our experimental results showed that cisplatin significantly decreased mitochondrial membrane potential and stimulated caspase 3 activity in a concentration - dependent fashion in APL cells [Fig. 5B–5C]. Our confocal imaging results also showed that JC-1 monomer (green color) expression increased concentration - dependently in cisplatin-treated APL cells compared to control [Fig. 6A].

### Cisplatin modulates stress signaling

**Figure 7:**
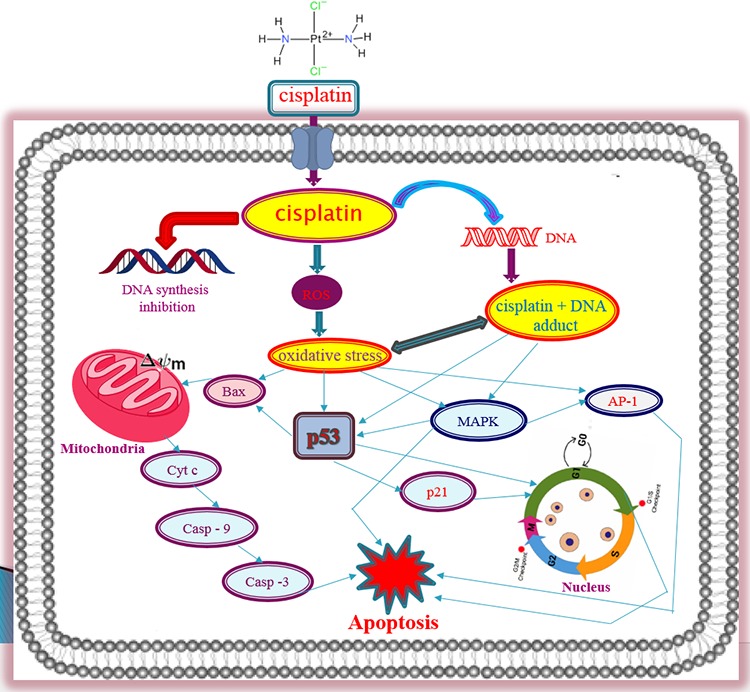
Molecular mechanisms of Cisplatin-induced toxicity in APL cells The overall molecular mechanisms of cisplatin cytotoxicity in APL cells involve many steps. In brief, cisplatin enters into the cell and interacts with DNA to form DNA adduct and also inhibits DNA synthesis. It produces ROS and creates oxidative stress inside the cell. DNA adduct formation and oxidative stress in APL cells cause the activation of p53 signaling and AP-1 phosphorylation leading to a change in mitochondrial membrane potential and cell cycle arrest in cells. This leads to cytochrome C release from the mitochondria, which activates caspase 9 and a series of complexes leading to activation of caspase 3 and apoptosis. Cisplatin cytotoxicity also modulates MAPK signaling pathway and overall leads to cancer cell death [7].

p38MAPK signaling molecules are very sensitive for any kinds of stress inside the cells. To investigate cisplatin involvement in stress signaling in APL cells, we evaluated the phophorylation level of p38 MAPK pathway molecules in both control and cisplatin treated cells. Our results indicated that cisplatin decreased the phosphorylation level of p38 MAPK molecules in a concentration - dependent manner [Fig. 6B]. Overall, cisplatin-induced cytotoxicity in APL cells is modulated through a series of important molecular mechanisms of action as shown in [Fig. 7].

## DISCUSSION

Cytotoxicity is a complex process mediated through DNA-adduct, oxidative stress, transcriptional factors and stress signaling leading to cell death. Previous studies have reported that cisplatin produces cytotoxic action by interacting with DNA and forming DNA-adduct, predominantly intra -strand crosslink adducts [6, 16] that lead to activation of various signaling events like p53, MAPK, etc, and finally culminating through apoptosis pathway [6, 10, 13, 17 and 18]. It has also been reported that cisplatin –induced cytotoxicity causes apoptosis by activation of p53 or p53-related protein, p73 [13]. Existing evidence suggests that approximately 1% cellular cisplatin interacts with DNA and forms DNA adduct [19]. This indicates that cisplatin induced cytotoxicity by other possible mechanisms in cancer cells. Earlier studies have pointed out that cisplatin inhibits cell proliferation by oxidative stress and apoptosis in HL-60 cells [11, 12]. But the detailed molecular mechanism of cytotoxic action of cisplatin in APL cells mostly remains unknown. Here, we have elucidated the molecular mechanisms of cisplatin cytotoxicity in APL cells. Our findings indicate that cisplatin inhibits APL cells proliferation (Fig. 1A–1C) in a concentration dependent fashion. It induces cytotoxicity in APL cells (Fig. 2A–2C) by interacting with DNA and forming DNA-adduct (Fig. 1D (i-vi)) in a concentration-dependent manner. Accumulating data have suggested that cisplatin produces reactive oxygen species (ROS) [18, 20] and induced oxidative stress [12] through lipid peroxidation product, MDA production by reducing cellular GSH level [21]. Our data presented here reveal that cisplatin produces reactive oxygen species (Fig. 3A) and induces oxidative stress through increasing lipid peroxidation (MDA level) by reducing GSH content in APL cells in a concentration - dependent fashion (Fig. 3B–3C). Several groups have provided evidence that cisplatin produces clastogenic (DNA damaging) effect that significantly contributes to its cytototoxic action by forming DNA-adduct in various cancer cells [6, 13, 16, and 18]. We found that cisplatin induces clastogenic effect very significant in a concentration - dependent fashion in APL cells both in TUNEL assay (Fig. 3D) and comet assay (Fig. 3E–3F). It has been reported from other studies that cisplatin induces cytotoxicity through activation of p53 [6, 13] and transcriptional factor, AP-1 and increased expression of c-jun in APL cell line [6, 10]. Many research groups have reported that p53 arrests cell cycle in cancer cells [2, 6 and 22] through activation of p21 [6, 13]. Our findings indicate that cisplatin produces cytotoxicity through causing cell cycle arrest by activation of p53, p21, down regulation of cyclins and cyclin dependent kinases {cdks} (Fig. 4A) in APL cells. We also found that cisplatin stimulates the expression and phosphorylation of AP-1 and its component, c-Jun at ser 63 & 73 involved in cell cycle arrest and apoptosis in APL cells (Fig. 4B). Cisplatin –induced cytotoxicity was also reduced the expression level of cell cycle proliferation marker, Ki67 in a concentration - dependent fashion in APL cells (Fig. 4C).

Several studies have demonstrated that cisplatin –induced cytotoxicity culminates in the activation of apoptosis in cancer cells [6, 13, 18]. It produces apoptosis through a mitochondrial membrane potential change and caspase 3 activation [23, 24]. Our results support these findings showing that cisplatin induces apoptosis in APL cells through changing mitochondrial membrane potential by up - regulation of pro-apoptotic protein, Bax and down-regulation of anti-apoptotic protein, Bcl-2 leading to formation of transition pores that helped to release cytochrome C into the cytoplasm. Cytochrome C activates caspase 9 and a series of signaling cascade leads to the activation of caspase 3 and apoptosis in APL cells (Fig. 5A–5C). It also activates JC-1 monomer expression in a concentration dependent manner in APL cells (Fig. 6A). Other studies have reported that cisplatin induced cytotoxicity was modulated by p38 MAPK signaling pathway, which coordinated AP-1 components phosphorylation or expression and apoptosis [6, 17 and 25]. Our findings also demonstrated that cisplatin modulates MAPK signaling pathways in APL cells in a concentration dependent manner (Fig. 6B).

Taken together, it can be concluded from our research that cisplatin inhibits APL cells proliferation through a cytotoxicity, which is mediated by DNA adduct formation, induction oxidative stress and cell cycle arrest, activation of transcriptional factors, intrinsic pathway of apoptosis, and modulation of MAPK signaling cascade [Fig. 7]. By elucidating the cytotoxic action of cisplatin in APL cells, we have provided new insights into the molecular targets, and a rational basis for drug designing for a more prominent APL chemotherapy in the future.

## MATERIAL AND METHODS

### Cell line and culture

Three APL cell lines were used in this study including HL-60, NB4 and KG-1a cells. The cells were purchased from the American Type Culture Collection (Manassas, VA), and maintained at 37°C in an atmosphere of 5% CO_2_ and 95% air according to standard procedures.

### Chemicals and reagents

Cisplatin was purchased from Abcam (Cambridge, MA) and Sigma Aldrich (St. Louis, MO) and thymidine (methyl-^3^H) was obtained from MP Biomedical (Santa Ana, CA). Mitochondrial isolation kit, caspase assay kit, protease inhibitor and glutathione assay kit were obtained from Sigma-Aldrich (St. Louis, MO). Phosphospecific anti-Erk, anti JNK, anti-P38MAPK, anti-cytochrome C, anti-Bax and anti-Bcl2 were purchased from Cell Signaling Technology (Danvers, MA). Lipid peroxidation kit and caspase 3 kit were obtained from Abcam (Cambridge, MA). Hoechst 33342, Alexa fluor 568 and Alexa fluor 568 were purchased from Life Technologies (Grand Island, NY).

### Cell proliferation assay

APL cells were exposed to various concentrations (0, 5, 10, 20, 40, and 80 μM) of cisplatin in triplicate and labeled with thymidine methyl- ^3^H. Cells were harvested and the radioactivity measurement was performed using a liquid scientillation analyzer as previously described [26].

### Cytotoxicity assay

APL cells were grown in absence or presence to various concentrations (0, 5, 10, 20, 40, and 80 μM) of cisplatin for 48 hrs inside an incubator maintaining standard temperature (37^°^C) and 5% CO_2_ level. After incubation, the cells were centrifuged and the supernatant was collected from all samples. LDH release in control and cisplatin treated APL cells supernatant was measured by following promega non-radioactive cytotoxicity assay technical bulletin protocol (Cat # G1780). Experimental data were presented as % of LDH release in cisplatin - treated APL cells at different concentrations (0, 5, 10, 20, 40, and 80 μM) compared to total LDH released in control cells.

### Measurement of reduced GSH

Acute leukemia cells were grown in presence or absence of cisplatin and the GSH content inside the cytoplasm was measured following a previously published protocol [27].

### Lipid peroxidation assay

APL cells were treated with or without cisplatin and lipid peroxidation was evaluated by measuring malondialdehyde (MDA) levels using the lipid peroxidation assay kit (Abcam) as previously described [27].

### TUNEL assay

The TUNEL assay is very sensitive technique to analyze DNA damage in tissue section or cells. In brief, acute leukemia cells were grown in presence or absence of cisplatin, and DNA damage was analyzed by immunochemistry and confocal imaging using Promega DeadEnd™ Fluorometric TUNEL System Technical Bulletin (Cat# G3250) or as previously described [28]. Both untreated/control and cisplatin treated APL cells were attached on poly-l-lysine coated chambered slide and fixed with 4% paraformaldehyde at 4^°^C for 25 min. Fixed cells were permeabilized with 0.2% triton X-100 in PBS for 5 min. Slides were washed with PBS two times and equilibrated in equilibration buffer for 10 min at room temperature. 100 μl rTdT incubation buffer were added on each slide and incubated at 37^°^C for one hour. After incubation, the reaction was terminated by dipping slides in 2X SSC for 15 min and washed 2–3 times with PBS. The slides were stained with DAPI and mounted by anti-fade solution with coverslip and nail polish. After drying of slides, confocal imaging was performed using the fluoview confocal microscopy system (Olympus company).

### Single cell gel electrophoresis (Comet) assay

APL cells were cultured in presence or absence of cisplatin and DNA damage was analyzed by performing a very sensitive alkaline comet assay as previously described [2, 29]. In brief, agarose was prepared through melting in a boiling water bath and allowing it to return to room temperature. The cells were mixed with the melted agarose in a 1:10 ratio. Approximately 50 μL of the mixture of agarose and cells were placed on comet slides, and the agarose was solidified at 4°C for 45 min. After incubation, the slides were placed in a lysis solution at 4°C for 30 min to lyse the embedded cells in the agarose. The excess lysis solution was removed from the slides and placed in an alkaline solution to denature the DNA for 40 min at room temperature. Later, the slides were subjected to TBE (Tris borate EDTA buffer) electrophoresis for 30 min with 21 volt current between the two electrodes. Then the slides were fixed with 70% ethanol for 5 min, followed by SYBR green staining. The stained slides were examined using an epifluorescence microscope (Olympus BX51 TRF, USA). The data were analyzed with DNA damage analysis software (Loats Associates Inc., USA). The control comet slides were prepared along with the test comet slides under yellow light.

### Western blotting analysis

After treatment of APL cells to various concentrations of cisplatin, protein lysate were prepared in RIPA buffer with sonication and centrifugation. An equal amount (40 mg) of protein from control or treated cells was loaded per lane on a 10% SDS-PAGE gel, transferred into nitrocellulose membrane and analyzed by western blotting for each specific protein of interest using its specific antibody as described previously [30]. The band intensities were quantified using Image J (National Institutes of Health).

### Caspase -3 activity assay

Caspase-3 activity was measured in cytosolic fraction of control and cisplatin exposed APL cells, using a kit purchased from Sigma (Sigma, St. Louis, MO, USA). In brief, cytosolic fraction of cells from both control and cisplatin – treated cells was prepared as described earlier [31]. Equal amount of cytosolic proteins were used for the assay of caspase 3 activity. Cytosolic protein (50 mg) was mixed in a microtiter plate with assay buffer and caspase specific substrates (Ac-DEVD-pNA for caspase-3). After 4–16 h incubation at 37°C, the absorbance of pNA released as a result of caspase-3 like activity was measured at 405 nm in a microtiter plate reader as described in technical bulletin. The absorbance of negative control (assay buffer substrate) was subtracted from specific values. Mean values of triplicate measurements were computed.

### Measurement of mitochondrial membrane potential (Δψm)

The integrity of inner mitochondrial membrane was examined by observing the changes in the potential gradient across the membrane. This was achieved by measuring the uptake of the cationic carbocyanine dye, JC1 into the matrix. Mitochondria were isolated from control and cisplatin-treated APL cells using mitochondria isolation kit (Sigma, St. Louis, MO, USA). Isolated mitochondria were incubated with 2 μl JC1 stain (from stock 1 mg/ml) and 950 μl JC1 assay buffer for 10 min in dark at 25°C. The fluorescence of each sample (total assay vol. 1 ml) was recorded using a Perkin Elmer LS50B spectrofluorometer (excitation 490 nm, slit, 5 nm; emission 590 nm, slit, 7.2 nm) [32].

### Immunocytochemistry and confocal imaging

APL cells (1 × 10^5^) were cultured in presence or absence of cisplatin and attached on poly-L-lysine coated slide. Cells were fixed by using 3% paraformaldehyde and permeablized with 0.2% NP-40 containing 0.5% glycine. After blocking with 4% BSA, fixed cells were incubated overnight with Ki-67 antibody (dilution, 1:100) (cat# 33-4711) or cisplatin modified DNA adduct antibody (cat# ab103261) from Life Technology or Abcam company at 4°C. After incubation, cells were washed with PBS three times and tagged with secondary antibody (anti-mouse fluorescein) for one hour at room temperature followed by Hoechst 33342(dilution, 1:2000) staining for 7 min. Slides were washed with PBS and paste coverslip using prolong gold antifade reagent. After drying, slides were imaged by confocal microscopy (Olympus company, Center valley, PA).

### Statistical analysis

Experiments were performed in triplicates. Data were presented as means ± SDs. Where appropriate, one-way ANOVA or student paired *t*-test was performed using SAS Software available in the Biostatistics Core Laboratory at Jackson State University. *p*-values less than 0.05 were considered statistically significant.
